# Relationship between executive functions and academic performance among Moroccan middle school students

**DOI:** 10.1590/1980-57642020dn14-020014

**Published:** 2020

**Authors:** Mounir Bouzaboul, Abdeslam Amri, Zakaria Abidli, Hassan Saidi, Noureddine Faiz, Rabea Ziri, Ahmed Ahami

**Affiliations:** 1 Cognitive Behavioral Neuroscience and Applied Nutrition Team, Faculty of Sciences, University of Ibn Tofail, Kenitra, Morocco.; 2 Genetics and Biometrics Laboratory, Faculty of Sciences, University of Ibn Tofail, Kenitra, Morocco.

**Keywords:** executive functions, learning difficulties, middle school, Morocco, funções executivas, dificuldades de aprendizagem, ensino médio, estudantes, Marrocos

## Abstract

Executive functions (EF) play a central role in the development of social and cognitive skills and academic learning.

**Objective:**

For this reason, this study aims to determine the relationship between executive functions and academic performance among middle school students in the Middle Atlas of Morocco.

**Methods:**

This study focuses on 137 middle school students studying at four colleges located in the Middle Atlas of Morocco. The sample studied was divided into two groups: the first consisting of students with learning difficulties; and the second considered a control. To assess EF, three tests were administered to learners in both groups (Tracking Test, Stroop Test and Number Span Test).

**Results:**

In the sample, average age of the learners was 14.5±1.3 years and sex ratio was balanced. The students with learning difficulties had lower performance on tests measuring cognitive flexibility, inhibitory processes and working memory compared to the control group.

**Conclusion:**

From these results, it can be concluded that students with learning disabilities performed poorly on the three basic components of executive functions. Therefore, thorough neuropsychological diagnosis would be desirable to identify learners who may have cognitive or behavioural disorders and allow adequate intervention to improve their executive functions and subsequently their academic success.

## RESUMO.

As funções executivas (FEs) desempenham um papel central no desenvolvimento de habilidades sociais e cognitivas e no aprendizado acadêmico.

**Objetivo:**

Por esse motivo, nosso estudo teve como objetivo determinar a relação entre FEs e desempenho acadêmico entre estudantes do ensino médio no Atlas Médio de Marrocos.

**Métodos:**

Nosso estudo se concentrou em 137 alunos do ensino médio que estudavam em quatro colégios localizados no Atlas Médio de Marrocos. A amostra estudada foi dividida em dois grupos, o primeiro composto por alunos com dificuldades de aprendizagem e o segundo considerado como controle. Para avaliar os FEs, três testes foram administrados aos alunos de ambos os grupos (Testes de Trilhas, Teste de Stroop e Teste de Extensão de Dígitos).

**Resultados:**

A idade média dos alunos em nossa amostra foi de 14,5±1,3 anos, com uma relação entre gêneros equilibrada. Em nosso estudo, os alunos com dificuldades de aprendizagem apresentaram desempenho inferior nos testes que mediram flexibilidade cognitiva, processo inibitório e memória de trabalho em comparação ao grupo controle.

**Conclusão:**

Do ​​nosso resultado, podemos concluir que alunos com dificuldades de aprendizagem apresentam um desempenho fraco nos três componentes básicos das FEs. Por esse motivo, seria desejável um diagnóstico neuropsicológico completo, para identificar alunos que possam ter distúrbios cognitivos ou comportamentais para uma correção adequada para melhorar seus FEs e, posteriormente, seu sucesso acadêmico.

Executive functions (EF) are defined as the set of processes by which an individual intentionally regulates their thought and actions in order to achieve goals.^1^ Based on neuropsychological and neurophysiological studies, it is widely accepted that the prefrontal cortex plays a key role in supporting executive functions.^2^ However, the different functionalities of executive functions seem to recruit different parts of the frontal cortex, as well as other regions of the brain.^3^ Recent studies have divided EFs into major components, three of which have been differentiated: inhibition, working memory (WM) and cognitive flexibility.^4^^,^ 5 Many studies have shown the central role of EF in the development of social and cognitive skills and in academic learning, especially among preschool and school-aged children.^6^^,^ 7 Empirical studies show that the presence of deficits at the EF level contributes to the emergence of academic difficulties, which hinder learners’ success.^8^^,^ 9 Although there is a large body of research on the relationship between EF and academic success during childhood and early childhood, fewer studies have examined the correlations between EF and academic performance during adolescence,^10^ despite the importance of EF during this period for effective daily functioning.^8^^,^ 9 ^-^ 11 This prompted the present study to establish the relationship between executive functions and academic performance among a sample of middle school students from the Middle Moroccan Atlas.

## METHODS

### Participants

A total of 137 middle school students studying in four colleges, all belonging to the province of Khenifra located in the Middle Moroccan Atlas, were investigated. The average age of the learners was 14.5±1.3 years with a maximum age of 16 years and minimum of 12 years.

### Methodology and study instruments

The study of learners’ files, their academic performance and the opinions of teachers allowed students with learning difficulties to be identified. The sample studied was divided into two groups: the first consisting of students with learning difficulties (n=69); and the second comprising students without learning difficulties (n=68), with the latter group serving as the control in this study. To assess EF, three tests were administered to learners in both groups.

#### Trail Making Test (TMT)

This test was designed by (Reitan and Wolfson, 1985) to assess cognitive flexibility, visual and visual motor exploration. The TMT has proven popular with clinical psychologists because of its advantages, in particular its simplicity of administration and speed. This test consists of two parts, Part A of the TMT entails linking a series of increasing numbers from 1 to 25 by selecting, at each point, the relevant number from among the 25 possible items. In Part B of the TMT, the subject must handle two series alternately: a series of numbers and a series of letters (1-A-2-B-3-C...13). It is therefore a question of planning in parallel, but alternately, two automated series without them interfering with each other, by permanently activating the relevant sequence, while temporarily inhibiting the second. Part B is more complex than Part A, and allows the evaluation of shifting (Bowie & Harvey, 2006).^12^

#### Stroop Test

The objective of this test is to evaluate the inhibitory process, and is among the most commonly used in psychological and neuroscientific research and practice.^13^ In this test, three A4 format cards are used:

- The first card (A) has a series of words written in black ink and names 4 colors (green, yellow, red, blue) randomly arranged in ten rows of five words.
- The second card (B) represents a sequence of colored rectangles (green, yellow, red, blue) randomly arranged in ten rows of five rectangles.
- The third card (C) retains the characteristics of card A (with a new random arrangement) but the printing ink differs for each of the words. The color name is never printed using the named color.

For the final score, the number of items processed and the number of errors made by the learners for the three tests during 45 seconds were recorded, according to Desbrosses’ rating.^14^

#### Number span test

This is a subtest of the WISC III (Wechsler Intelligence Scale for Children) verbal scale to assess short-term verbal memory abilities and working memory in children. It entails determining the maximum number of digits that the child is able to repeat in the order in which they were stated: this is the digit span forward. When the number of digits that the child must repeat is in the opposite order to the one in which they were stated: it is the digit span backward. This allows an estimation of working memory capacities.^15^

### Statistical analyses

For the statistical analysis, descriptive statistics and analytical statistics were used. Descriptive statistics were expressed as percentages, means and standard deviations of the quantitative variables studied. For the analytical statistics, Student’s *t*-test was used to compare the means of the variables studied in the three tests between the group of students with learning difficulties and the control group.

## RESULTS

### Socio-demographic and academic profile of sample studied

This study involved 137 learners studying in Khenifra province located in the Middle Atlas of Morocco. The average age of the learners was 14.5±1.3 years, with a maximum age of 16 years and a minimum of 12 years. In this study, the sex ratio was balanced (53% boys). The learners in the sample were drawn from two settings: 43% from urban areas; and 57% from rural areas (Table 1).

**Table 1:** Socio-demographic parameters of the learners in our sample.

		Size	Percentage
Gendre	Male	73	53%
	Feminine	64	47%
Environment	Urban	59	43%
	Rural	78	57%
Age	14.5±1.3 ans/ (Max=16 ans ; Min=12 ans)		

Of the 137 learners in the sample studied, 50.4% had poor academic performance and 49.6% had no learning difficulties.

In the present study, there was a significant association between age and academic performance (P-value <0.05) with a high percentage in learners aged >15. Regarding gender, a correlation between gender and academic performance (P-value <0.05) was noted, with a higher percentage in male learners (Table 2).

**Table 2:** Distribution of two groups according to age, gender and school environment.

Variable		Learning difficulties group (n=69)	Control group (n=68)	P-value
Age (years)	[12-13]	14 (40%)	21(60%)	<0.05*
	[14-15]	34 (49%)	36 (51%)	
	>15	21 (66%)	11(34%)	
Gendre	Feminine	27 (42%)	37(58%)	<0.05*
	Male	42 (58%)	31 (42%)	
School environment	Urban	33 (56%)	26 (44%)	n.s
	Rural	36 (46%)	42 (54%)	

* Significant: P-value <0.05; n.s: Is not significant P-value >0.05.

#### Cognitive flexibility

The average scores of students in the learning difficulties group and the control group on the two TMT tasks were compared using Student’s t-test (Table 3).

**Table 3:** Comparison of average scores in the two TMT tasks between the learning difficulties group and the control group.

	Group of learners	N	Average (s)	Standard deviation	t	P-value
TA: Time to complete TMT task A	learning difficulties group	69	43.7	13.7	2.4	0.0***
	control group	68	37.2	17.0		
TB: Time to complete TMT task B	learning difficulties group	69	125.6	46.8	3.8	0.0***
	control group	68	97.4	37.5		
TB - TA	learning difficulties group	69	81.8	47.7	3.8	0.0***
	control group	68	60.2	32.6		

*** P- value < 0.0001 highly significant.

Results showed higher averages in the learning difficulties group in terms of the time taken to complete tasks A and B and the time difference TB-TA (TA=43.7±13.7; TB=125.6±46.8; TB-TA=81.8±47.7, respectively) compared to means for the control group (TA=37.2±17.0 ; TB=97.4±37.5 ; TB-TA =60.2±32,6).

Similarly, Student’s t-test showed a highly significant difference between the two groups for scores on the two TMT tasks A and B and the time difference between the two tasks [(t=2.4, p<0.000) for task A; (t=3.8, p<0.000) for task B, and (t=3.8, p<0.000) for the time difference between the two tasks, respectively].

#### Inhibition

The use of Student’s *t*-test allowed comparison of the average scores recorded and the average errors made by learners in both groups upon administering the Stroop test (Table 4).

**Table 4:** Comparison of the average scores recorded by learners in the two groups in the three tasks of the Stroop test and the errors made by learners in administering the test.

	Group of learners	N	Average (s)	Standard deviation	t	P-value
Reading task	learning difficulties group	69	82.9	10.9	-5.1	0.0***
	control group	68	93.7	13.7		
Denomination Task	learning difficulties group	69	44.4	8.3	-6.5	0.0***
	control group	68	54.9	10.4		
Interference task	learning difficulties group	69	36.3	6.4	-3.5	0.0***
	control group	68	40.3	6.9		
Errors in the reading task	learning difficulties group	69	1.1	1.1	2.3	0.0***
	control group	68	.6	1.1		
Errors in the denomination task	learning difficulties group	69	1.8	1.1	3.7	0.0***
	control group	68	1.0	1.1		
Errors in the Interference task	learning difficulties group	69	2.9	1.6	3.6	0.0***
	control group	68	1.9	1.5		

*** P- value < 0.0001 highly significant.

Results showed low average scores in the learning difficulties group for the three Stroop test tasks (82.9±10.9 for reading task; 44.4±8.3 for naming task and 36.3±6 for interference task, respectively) compared to average score for the control group (93.7±13.7 for first task; 54.9±10.4 for second task and 40.3±6.9 for third task). Similarly, a comparison of the average errors made by learners showed that students with learning difficulties made more errors (1.1±1.1 for reading task; 1.8±1.1 for naming task and 2.9±1.6 for interference task) compared to average scores for the control group (0.6±1.1 for first task; 1.0±1.1 for second task and 1.9±1.5 for third task).

Regarding the results of Student’s *t*-test, there was a highly significant difference between the two groups for the average total scores of the learners on the three Stroop tasks [(t= -5.1, p<0.000) for item score in reading task; (t= -6.5, p<0.000) for item score on naming task; (t= -3.5, p<0.000) for item score of on interference task and (t= -4.7, p<0.000) for interference score, respectively]. Similarly, there was a highly significant difference between the two groups in relation to errors made by learners on the three tasks of the Stroop test [(t=2.3, p<0.000) for errors on reading task; (t=3.7, p<0.000) for errors on naming task; and (t=3.6, p<0.000) for errors on interference task].

#### Working memory

The mean scores on the forward and reverse span of the groups of learners with learning difficulties and the control group were compared using Student’s *t*-test (Table 5).

**Table 5:** Comparison of the mean scores in the number span test between the learning disabled group and the control group.

	Group of learners	N	Average	Standard deviation	t	P-value
Direct span	Learning difficulties group	69	3.7	.6	-3.4	0.0***
	Control group	68	4.1	.5		
Inverted span	Learning difficulties group	69	2.0	.5	-3.6	0.0***
	Control group	68	2.3	.5		

*** P- value < 0.0001 highly significant.

Results showed low average scores in the learning difficulties group on the forward and backward span (3.7±0.6 and 2±0.5, respectively) compared to the average scores for the control group (4.1±0.5 and 2.3±0.5).

Analysis of the results of the Student test revealed a highly significant difference between the two groups in mean scores on the forward and backward digit span tests [(t= -3.4, p<0.000) and (t= -3.6, p<0.000) respectively].

## DISCUSSION

The use of neurocognitive tests in this study aimed to highlight the relationship between executive functions and academic performance in a sample of middle school students. The study results showed that students with learning difficulties had low scores compared to the control group subjects on the three basic components of EF, namely, mental flexibility, the inhibitory process and working memory. These results are in harmony with previous work, where EF involve the creation and implementation of a plan, self-monitoring, and cognitive flexibility - skills that are an important element of academic success.^5^^-^ 10 Similarly, learners with more developed EFs are often the most successful in school and university, and have fewer behavioural problems.^16^ Academic success is intimately linked to learner performance in different domains such as mathematics, science, literacy (reading and writing), oral communication and social interactions, and these domains are influenced by the three basic components of EF.^17^

Cognitive flexibility or “switching”, is defined as the ability to change tasks or mental strategy and move from one cognitive operation to another^18^ and cited as the most complex executive component because it is underpinned by several executive processes.^1^^-^ 5 Students with learning difficulties had low scores compared to their control group peers, which leads us to conclude that students with learning disabilities perform less well in terms of cognitive flexibility. These results corroborate the work of several researchers in the field of mathematics, who have demonstrated that the learner must be flexible in moving from one strategy to another, for example, from a strategy of recovery, decomposition or transformation to a strategy of solving arithmetic problems.^19^ Similarly, cognitive flexibility explains variations in reading and writing performance among primary school learners, according to other researchers.^19^

Inhibitory control is one of the main components of executive functions and has been described both as the suppression of a pre-potent response and as interference control.^20^ Our study results showed that students with learning difficulties scored low on the Stroop test, a gold standard for assessing cognitive inhibition skills, compared to the control group subjects.^4^ This conclusion is in line with previous investigations showing that the inhibitory process is a crucial factor in academic success, including mathematical skills and the acquisition of reading among primary school children (Kamza, 2017).^21^ Other work has highlighted the association between inhibition and success in English, mathematics and science among learners aged 11-12 years (St Clair-Thompson & Gathercole, 2006).^22^ Similarly, inhibition ability is considered necessary for the active suppression of immature strategies, and information not relevant to the task during a mathematical problem according to several researchers.^23^

With regard to working memory (WM), defined as the ability to retain and mentally manipulate information for a limited period of time,^24^ students with learning difficulties had poor performance on visuospatial WM tasks compared to their peers in the control group. These results are consistent with other research that has associated learner performance on tasks requiring visuospatial working memory with performance in English, mathematics and science.^20^ Other studies have shown the existence of specific associations between WM and mathematics and reading performance.^10^ A growing number of studies have indicated that the updating of working memory can be closely related to mathematical learning and success.^19^^-^ 21

The results of this study revealed that the academic success of middle school students was associated with executive function performance. Exploring programs designed to promote EFs and to make better use of the strategies of these functions is of primary importance, given that several studies have correlated improvement in the use of EFs with academic success among children who have benefited from these programs.^5^^-^ 10

In conclusion, the neurocognitive tests used indicate that the performance of middle school students with learning difficulties in the three basic components of executive functions was lower compared to the control group subjects, as the students without learning difficulties scored higher on cognitive flexibility, the inhibitory process and working memory. To further elucidate these associations, a thorough neuropsychological diagnosis would be desirable to identify learners who may have cognitive disorders classified as “Dys”, attentional deficit or behavioural disorders. This would allow for adequate interventions to improve their executive functions and subsequently their academic success.
